# Bi-layer Channel AZO/ZnO Thin Film Transistors Fabricated by Atomic Layer Deposition Technique

**DOI:** 10.1186/s11671-017-1999-7

**Published:** 2017-03-24

**Authors:** Huijin Li, Dedong Han, Liqiao Liu, Junchen Dong, Guodong Cui, Shengdong Zhang, Xing Zhang, Yi Wang

**Affiliations:** 1 Institute of Microelectronics, Peking University, 100871 Beijing, China; 2 Shenzhen Graduate School, Peking University, 518055 Shenzhen, China

**Keywords:** Thin-film transistor, ALD, Bi-layer channel, ZnO, AZO

## Abstract

This letter demonstrates bi-layer channel Al-doped ZnO/ZnO thin film transistors (AZO/ZnO TFTs) via atomic layer deposition process at a relatively low temperature. The effects of annealing in oxygen atmosphere at different temperatures have also been investigated. The ALD bi-layer channel AZO/ZnO TFTs annealed in dry O_2_ at 300 °C exhibit a low leakage current of 2.5 × 10^−13^A, *I*_on_/*I*_off_ ratio of 1.4 × 10^7^, subthreshold swing (SS) of 0.23 V/decade, and high transmittance. The enhanced performance obtained from the bi-layer channel AZO/ZnO TFT devices is explained by the inserted AZO front channel layer playing the role of the mobility booster.

## Background

Oxide thin-film-transistors (TFTs) have a growing need for the development of transparent displays, flexible electronics, and organic light-emitting diodes due to their excellent electrical and optical properties even at low deposition temperatures [1–3]. While a great number of various deposition techniques were reported on oxide thin films, the main deposition methods for oxide active layers in TFTs are based on physical vapor deposition (PVD), such as magnetron sputtering [4, 5], pulsed laser deposition [1], and evaporation [6]. However, PVD technique has some problems such as non-reproducibility and non-uniformity for thin film composition in the growth of multicomponent oxide films, which hinder the mass production of the TFTs based on multicomponent oxides [7].

Atomic layer deposition (ALD) is a gas-phase thin film deposition technology characterized by the alternate exposure of chemical species with self-limiting surface reactions, providing extremely high uniformity, as well as thickness and composition control for the deposition of various oxides, nitrides, and sulfides [8–11]. Especially, ALD can produce high quality films at a relatively low temperature, making it compatible with both glass and plastic transparent substrates [12]. Furthermore, oxide thin films processed by ALD are compatible not only with planar device architecture, but also with emerging 3D device architectures because ALD is capable of depositing conformal and uniform thin films on a wide range of substrates and geometries [13]. The material development for active layers grown by ALD is a key issue for the fabrication of TFTs based on all ALD processes. Recently, Wang YH et al. reported the effects of post-annealing on the performance of ALD ZnO/Al_2_O_3_ thin-film transistors [14]. Kwon S et al. reported that the processing temperatures have a huge impact on the characteristics of ALD ZnO thin film transistors [15]. Ahn CH et al. reported Al doped ZnO channel layers TFTs with improved electrical stability fabricated by atomic layer deposition at a relatively low temperature [7]. As advanced architecture for high performance TFTs, double channel devices have been widely investigated [16]. In particular, double channel structure is a simple and an effective method for optimizing the carrier concentrations and channel resistivity, leading to higher on-state current and mobility [17]. For example, Wang SL et al. reported high mobility indium oxide/gallium oxide bi-layer structures deposited by magnetron sputtering [18]. Kim SI et al. reported high performance ITO/GIZO double active layer TFTs formed by a radio frequency magnetron sputtering [19]. While double channel TFTs fabricated by sputtering were reported previously, the applications of the double-channel devices deposited by ALD have rarely been studied to date.

In this paper, we introduce the bi-layer channel AZO/ZnO TFTs fabricated using atomic layer deposition process at a relatively low temperature. The properties of ZnO, AZO, and bi-layer AZO/ZnO films were characterized by microstructure, crystal structure, and optical analysis techniques. The influences of annealing treatment for bi-layer channel AZO/ZnO TFTs have been discussed.

## Methods

The single-layer ZnO and bi-layer AZO/ZnO films were deposited on SiO_2_ (50 nm)/p++ − Si substrates by atomic layer deposition (ALD) at 125 °C. Deionized water (DW), diethylzinc (DEZn), and trimethylaluminium (TMA) precursors were used as the sources for oxygen, aluminum, and zinc, respectively. N_2_ was employed as the purging gas with a flow rate of 20 sccm. The pulse/purge times for Zn, Al, and O sources are 40, 20, and 20 ms/25 s, respectively.

**Fig. 1:**
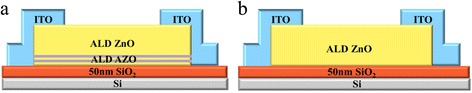
Cross-sectional schematic of the **a** bi-layer channel AZO/ZnO TFT device and **b** ZnO TFT device

As for bi-layer channel AZO/ZnO TFTs, AZO film [ZnO(19 cycles)/Al_2_O_3_(1 cycle)/ZnO(19 cycles)/Al_2_O_3_(1 cycle)] was deposited on the top of SiO_2_ as the front channel layer. Subsequently, 260 cycles ZnO was formed in order to fabricate the back channel layer. The bi-layer channel was defined by lift-off technique. Finally, the ITO source and drain regions were deposited by radio frequency (RF) sputtering in pure Ar atmosphere. Single-layer ZnO TFTs were fabricated in a similar way except for the active layer which was formed by depositing 300 cycles ZnO. The cross-sectional schematic of the bi-layer channel AZO/ZnO TFT device and ZnO TFT device is shown in Fig. 1.

The crystal structure of ZnO, AZO, and bi-layer AZO/ZnO films was measured by X-ray diffraction (XRD, Rigako), and their surface morphology was evaluated by atomic force microscope (AFM). The optical transmittance spectra was analyzed to investigate the optical properties of ZnO, AZO, and bi-layer AZO/ZnO films. The electrical properties of the fabricated TFTs were measured using a semiconductor parameter analyzer (Agilent 4156C) at room temperature.

## Results and Discussion

**Fig. 2:**
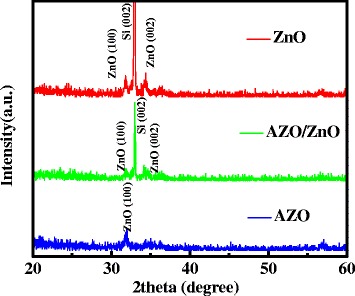
XRD patterns of AZO, ZnO, and AZO/ZnO films

Figure 2 demonstrates the X-ray diffraction (XRD) patterns of the corresponding ZnO, AZO, and bi-layer AZO/ZnO films deposited on the SiO_2_/Si substrate. Both of the ZnO and the bi-layer AZO/ZnO films show ZnO (100) and ZnO (002) peak, while only ZnO (100) reflection peak is presented in the AZO film, and the peak of ZnO (002) disappeared. This is attributed to the influence of stress arising from the difference in ionic radii of Zn and Al, leading to the degradation of crystallinity [20].

**Fig. 3:**
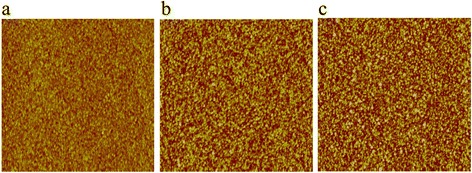
AFM images of **a** AZO, **b** ZnO, and **c** AZO/ZnO films

Figure 3 depicts the atomic force microscopy (AFM) images of ZnO, AZO, and bi-layer AZO/ZnO films on SiO_2_/Si substrate. The scanned area is 5 × 5 μm^2^ and is measured in the central regions of the film. The root mean square roughness (RMS) of ZnO, AZO, and bi-layer AZO/ZnO films is 1.4, 0.8, and 1.6 nm, respectively. All of the films exhibit low roughness of about 1 nm indicating that ALD technique can acquire smooth surface, which is conducive to obtain high performance devices.

**Fig. 4:**
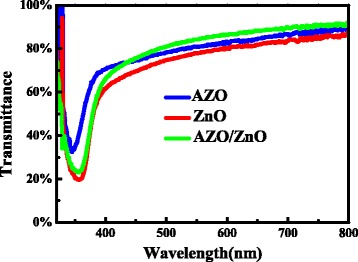
Optical transmittance spectra of AZO, ZnO, and AZO/ZnO films

**Fig. 5:**
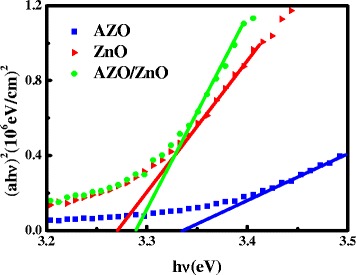
Plots of (αhν)^2^ versus hν for AZO, ZnO, and AZO/ZnO films

Figure 4 exhibits the optical transmission spectra of the ZnO, AZO, and bi-layer AZO/ZnO films. All the films show high transmittance in the visible region. The onset of fundamental absorption of ZnO causes the sharp fall in transmittance below 400 nm [21]. Absorption coefficient (α) can be extracted from the relationship [21],

1 $$ T\left(\uplambda \right) \approx {\left(1- R\left(\uplambda \right)\right)}^2{e^{-}}^{\upalpha \left(\uplambda \right) d} $$

where *d* is the film thickness, *T* is the transmittance, and *R* is the reflectance. Wurtzite structure ZnO has a direct band gap, and the absorption edge for a direct interband transition is given by [22]:

2 $$ {\left(\upalpha \mathrm{h}\upnu \right)}^2 = C\left(\mathrm{h}\upnu -{E}_{\mathrm{opt}}\right) $$

where *h* is Planck’s constant, *ν* is the frequency of the incident photon, and *C* is a constant for a direct transition. In order to approximate the optical band gap (*E*_opt_), we plot the (αhν)^2^ versus photon energy hν for ZnO, AZO, and bi-layer AZO/ZnO films, which is depicted in Fig. 5. The extrapolation of the curves’ straight-line segments toward the x-axis gives the optical band gap *E*_opt_ value. According to the results in Fig. 5, *E*_opt_ for ZnO, AZO, and bi-layer AZO/ZnO films is 3.27, 3.34, and 3.29 eV, respectively, which agrees well with bulk band gap of ZnO. This broadening in the band gap is mainly attributed to Moss–Burstein shift [23]. On the basis of the Moss–Burstein theory, the donor electrons occupy states at the bottom of the conduction band in heavily doped semiconductor. The valence electrons need extra energy to be excited to higher energy states in the conduction band because the Pauli principle prevents states from being doubly occupied, and optical transitions are vertical. Hence, doped zinc oxide films’ *E*_opt_ is broader than that of undoped zinc oxide films [24].

**Fig. 6:**
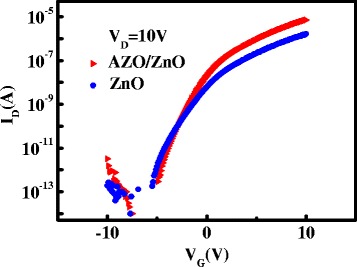
Transfer characteristics of ZnO TFTs and bi-layer channel AZO/ZnO TFTs

Figure 6 shows the transfer characteristics of the single-layer ZnO TFT and bi-layer channel AZO/ZnO TFT. The threshold voltage (*V*_th_) can be extracted by linear extrapolation of the *I*_D_^1/2^−*V*_G_ plot at saturation regions [3]. The single-layer ZnO TFT exhibits a *I*_on_/*I*_off_ ratio of 0.9 × 10^7^, mobility of 0.3 cm^2^/Vs, threshold voltage of −0.9 V, and SS of 0.42 V/decade. By comparison, bi-layer channel AZO/ZnO TFT exhibits better characteristics such as a low *I*_off_ of 2.9 × 10^−13^A, *I*_on_/*I*_off_ ratio of 2.4 × 10^7^, mobility of 0.6 cm^2^/Vs, threshold voltage of −1.2 V, and SS of 0.5 V/decade. As for bi-layer channel AZO/ZnO TFT, the active layer consists of AZO front layer and ZnO back layer. According to XRD patterns, ZnO films have stronger diffraction peaks than AZO and bi-layer AZO/ZnO films. From the results in Fig. 5, bi-layer AZO/ZnO films have a broader *E*_opt_ than ZnO films. Both of the characteristics can be the evidence suggesting the incorporation of Al into ZnO. Due to the doping of aluminum, AZO films have higher carrier concentrations. The AZO front channel layer inserted between the gate dielectric film and back channel layer plays the role of the mobility booster, leading to the increase of *I*_on_ and mobility [16].

**Fig. 7:**
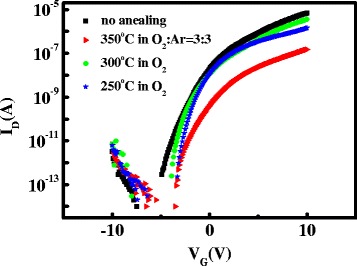
Transfer characteristics of bi-layer channel AZO/ZnO TFTs annealed in dry O_2_ at 300 and 250 °C, in dry O_2_:Ar = 3:3 at 350 °C for an hour

In order to improve the SS of device and to adjust the threshold voltage of TFTs, the bi-layer channel AZO/ZnO TFTs were annealed in oxygen atmosphere at different temperatures. Figure 7 pictures the transfer characteristics of bi-layer channel AZO/ZnO TFTs annealed in dry O_2_ at 300 and 250 °C, in dry O_2_:Ar = 3:3 at 350 °C for an hour. After annealing, all of the devices reveal a sharper SS and positive *V*_th_ shifts. The extracted electrical parameters of annealed devices are shown in Table 1. The bi-layer channel AZO/ZnO TFT which is annealed in dry O_2_ at 300 °C exhibits a superior performance such as SS of 0.23 V/decade, a low *I*_off_ of 2.5 × 10^−13^A, *I*_on_/*I*_off_ ratio of 1.4 × 10^7^, mobility of 0.4 cm^2^/Vs, and threshold voltage of −1.0 V. The promotion of SS after annealing treatment is mainly attributed to the reduction of defect density. The subgap density of states (DOS) is separated into the interface (*N*_it_) and the bulk (*N*_sg_) regions [25]. The effective interface trap state densities (*N*_it_) near/at the interface between the SiO_2_ and AZO are evaluated from the SS values [26]. By ignoring the depletion capacitance in the active layer, the *N*_it_ can be obtained from the expression [27]:

**Table 1:** The extracted electrical parameters of bi-layer channel AZO/ZnO TFTs with different annealing treatments

Annealing conditions	SS (*V*/dec)	*I*_on_/*I*_off_	*V*_th_ (*V*)	*μ* (cm^2^/V.s)	*I*_off_ (A)	*N*_it_ (cm^−2^)
No annealing	0.5	2.4 × 10^7^	−1.2	0.6	2.9 × 10^−13^	3.18 × 10^12^
Dry O_2_ at 300 °C	0.231	1.4 × 10^7^	−1.0	0.4	2.5 × 10^−13^	1.24 × 10^12^
Dry O_2_ at 250 °C	0.226	0.6 × 10^7^	−0.8	0.1	2.3 × 10^−13^	1.2 × 10^12^
Dry O_2_:Ar = 3:3 at 350 °C	0.166	0.6 × 10^7^	−0.4	0.01	1.0 × 10^−14^	0.77 × 10^12^

3 $$ {N}_{\mathrm{it}}=\left(\frac{\mathrm{SS}}{1\mathrm{n}10}\frac{q}{\mathrm{kT}}-1\right)\frac{C_{\mathrm{ox}}}{q} $$

where *q* is the electronic charge, *k* is the Boltzmann constant, *T* is the temperature, and *C*_ox_ is the gate capacitance density. The *N*_it_ value of bi-layer channel AZO/ZnO TFTs without annealing and with annealing in dry O_2_ at 300 °C is about 3.18 × 10^12^ and 1.24 × 10^12^ cm^−2^, respectively. It can be seen that annealing treatment decreases *N*_it_, leading to the improvement of SS. The *N*_it_ value of other devices annealed under different conditions is exhibited in Table 1.

**Fig. 8:**
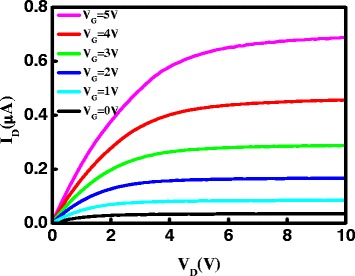
Output characteristics of bi-layer channel AZO/ZnO TFTs annealed in dry O_2_ at 300 °C for an hour

Under post-annealing in oxygen ambient, a portion of oxygen vacancies in the as-deposited AZO/ZnO thin film can be filled, and the carrier concentration decreases, resulting in the degradation of drain current [28]. Besides, it is a generally held view that a lower concentration of free electrons in active layer brings out a higher threshold voltage [29]. Therefore the extracted *V*_th_ of bi-layer channel AZO/ZnO TFTs increases after the post-annealing in oxygen atmosphere. Figure 8 depicts the output characteristics of the bi-layer channel AZO/ZnO TFT devices annealed in dry O_2_ at 300 °C. We believe that the characteristics of bi-layer channel AZO/ZnO TFTs can be enhanced by optimizing the thickness of AZO, ZnO, and the Al content of AZO.

## Conclusions

In summary, we have fabricated bi-layer channel AZO/ZnO TFTs via atomic layer deposition process at a relatively low temperature. The bi-layer channel AZO/ZnO TFTs exhibit a better performance than that of the single-layer ZnO TFTs. These results are attributed to the inserted AZO front channel layer serving as the mobility booster. In order to improve the SS of devices, bi-layer AZO/ZnO TFTs have been annealed in oxygen atmosphere at different temperatures. The results demonstrate that ALD bi-layer AZO/ZnO channel can be a promising candidate for the active layer of TFTs.
